# Phylogenetic and morphological reassessment of *Sporidesmium*-related taxa reveals *Lignosporidesmium* gen. nov. and *Xenosporidesmium lignicola* sp. nov.

**DOI:** 10.3897/mycokeys.142.216747

**Published:** 2026-10-29

**Authors:** Hong-Wei Shen, Kevin D. Hyde, Ying Wang, Lin Yang, Xi-Jun Su, Yun-Xia Li, Zong-Long Luo

**Affiliations:** 1 College of Agriculture and Biological Science, Dali University, Dali 671003, Yunnan, China College of Agriculture and Biological Science, Dali University Dali China; 2 Co-Innovation Center for Cangshan Mountain and Erhai Lake Integrated Protection and Green Development of Yunnan Province, Dali University, Dali 671003, Yunnan, China Center of Excellence in Fungal Research, Mae Fah Luang University Chiang Rai Thailand; 3 Center of Excellence in Fungal Research, Mae Fah Luang University, Chiang Rai 57100, Thailand Co‑Innovation Center for Cangshan Mountain and Erhai Lake Integrated Protection and Green Development of Yunnan Province, Dali University Dali China

**Keywords:** 12 new taxa, lignicolous freshwater fungi, morpho-phylogenetic, sporidesmium-like taxa, taxonomic revision

## Abstract

*Sporidesmiumsensu lato* comprises several phylogenetically divergent lineages. Although lineages such as *Distoseptispora* and *Xenosporidesmium* have been intensively studied, others remain poorly resolved owing to incomplete type material, limited population sampling, or the absence of molecular data. In this study, *Sporidesmium*-related taxa were investigated using a multigene phylogenetic framework based on combined LSU, ITS, *rpb*2, SSU, and *tef*1-α sequence data, which resolved seven distinct phylogenetic lineages. Based on integrative evidence from morphology and phylogeny, a new species, *Xenosporidesmium lignicola*, is described, and a new genus, *Lignosporidesmium*, is introduced to accommodate *L. tropicale* (≡ *Sporidesmium tropicale*). In addition, nine new combinations are proposed, including *Lylea lignicola*, *Xenosporidesmium bostenicum*, *X. dianchiense*, *X. distoseptatum*, *X. hongheense*, *X. lacustris*, *X. mucilaginosum*, *X. yangzonghaiense*, and *X. yilonghuense*. Furthermore, the phylogenetic structure and classification of *Sporidesmium*-related groups are comprehensively discussed from both morphological and multigene phylogenetic perspectives. Distinctive morphological characters, including conidiogenesis, septum type, and the presence or absence of sheaths or appendages, are evaluated in relation to their phylogenetic placements. The boundaries and relationships among *Ellisembia*, *Lylea*, *Sporidesmium*, *Xenosporidesmium*, and related historical and newly established taxa are critically assessed. However, the taxonomic positions of several lineages remain uncertain due to missing type materials or a lack of molecular data, highlighting the need for re-collection, detailed morphological re-examination, and the generation of fresh sequence data. Overall, the pronounced morphological diversity and limited molecular representation of many historically described species underscore the challenges in achieving a stable and comprehensive classification, emphasizing the necessity of integrative taxonomic approaches combining morphology, fresh collections, and multigene phylogenetic analyses.

## Introduction

Lignicolous freshwater fungi are a group that inhabit submerged woody substrates in diverse freshwater environments (Hyde et al. 2016a). They exhibit high species diversity and play crucial roles in nutrient cycling, energy flow, and the maintenance of biodiversity within freshwater ecosystems (Hyde et al. 2016a; Calabon et al. 2022, 2023). Yunnan Province is one of the most important research hotspots for these fungi, owing to its complex topography, varied climates, and abundant freshwater resources (Luo et al. 2019; Shen et al. 2022, 2025; Bao et al. 2023; Xu et al. 2025). Numerous taxa of lignicolous freshwater fungi from both lotic (running water) and lentic (standing water) habitats have been documented in Yunnan Province (Cai et al. 2002; Shen et al. 2025; Wang et al. 2025; Xu et al. 2025). These findings not only enrich the regional fungal inventory but also provide essential baseline data and valuable insights for understanding the diversity, ecology, and evolutionary relationships of freshwater fungi in the province (Luo et al. 2019; Shen et al. 2022; Bao et al. 2023).

*Sporidesmiumsensu lato* comprises hyphomycetous fungi characterized by solitary or gregarious conidiophores, mono- to polyblastic conidiogenous cells, and pale brown to dark brown, cylindrical, fusiform, obclavate, obpyriform, sometimes rostrate conidia, with hyaline apex and truncate base, with or without sheath (Ellis 1985; Wu and Zhuang 2005; Su et al. 2016; Liu et al. 2025). In recent years, with the extensive use of molecular sequence data, it has been possible to place taxa formerly in *Sporidesmium* or other genera in a natural classification system. These include *Distoseptispora (Distoseptisporaceae)*, *Ellisembia (Sporidesmiaceae)*, *Lomaantha (Chaetosphaeriaceae)*, *Xenosporidesmium (Xenosporidesmiaceae)* in *Sordariomycetes*, and *Pleotrichocladium (Melanommataceae)*, and *Neopeseudocercospora (Mycosphaerellaceae)* in *Dothideomycetes* (Braun et al. 2014; Su et al. 2016; Hernandez-Restrepo et al. 2017; Delgado et al. 2024; Liu et al. 2025). With advances in modern fungal taxonomy, phylogenetic analyses have become essential for delineating taxonomic boundaries (Jeewon and Hyde 2016; Chethana et al. 2021). When integrated with morphological evidence, they have substantially improved the resolution of several historically complex taxa (Hyde et al. 2021; Liu et al. 2024).

*Xenosporidesmiaceae* was established by Liu et al. (2025) to accommodate a lineage that is phylogenetically distinct from *Sporidesmiales* and *Distoseptisporales* within *Sordariomycetes* (Liu et al. 2025; Shen et al. 2025). The family comprises a single monogeneric lineage, *Xenosporidesmium*, with *X. aquaticivaginatum* (*Sporidesmium aquaticivaginatum*) as the type species. *Xenosporidesmium* is characterized by macronematous, mononematous, cylindrical, dark brown, septate conidiophores; holoblastic, monoblastic, integrated, terminal, determinate or with a single percurrent proliferation, cylindrical conidiogenous cells; and acrogenous, obclavate to rostrate, distoseptate, pale brown to olivaceous conidia paler toward the apex, and surrounded by a mucilaginous sheath or bearing an apical appendage (Su et al. 2016; Liu et al. 2019, 2025; Wang et al. 2024; Shen et al. 2025). Several species of *Xenosporidesmium* were previously placed in *Sporidesmium* because their morphology fit the broad concept of that genus (Hyde et al. 2016b; Liu et al. 2019; Bao et al. 2021; Wang et al. 2024; Shen et al. 2025). However, multiple phylogenetic analyses have shown that these species form a distinct, well-supported clade, separate from *Sporidesmiumsensu stricto* (Liu et al. 2025; Shen et al. 2025). To resolve this taxonomic incongruence, Liu et al. (2025) established *Xenosporidesmium* to accommodate this lineage. Currently, the genus comprises seven species: *X. caohaiense* and six species transferred from *Sporidesmium*, *viz*., *Xenosporidesmium aquaticivaginatum*, *X. filiforme*, *X. guizhouense*, *X. guttulatum*, *X. nujiangense*, and *X. olivaceoconidium* (Liu et al. 2025). These species have been reported primarily from freshwater habitats in China and Thailand (Hyde et al. 2016b; Liu et al. 2019; Bao et al. 2021; Wang et al. 2024; Shen et al. 2025).

During surveys of lignicolous freshwater fungi in Yunnan Province, China, two species of *Xenosporidesmium* were collected and identified. In conjunction with these findings, a comprehensive review of the relevant literature, together with newly generated multigene phylogenetic analyses, was conducted to revise and update the currently accepted species list of *Xenosporidesmium*. Furthermore, this study provides an integrative assessment of the phylogenetic structure and taxonomic framework of *Xenosporidesmium* and allied genera, including *Ellisembia*, *Lylea*, *Sporidesmium*, as well as other recently proposed taxa, based on combined morphological characters and multigene phylogenetic evidence. The species identifications and taxonomic interpretations proposed herein are grounded in careful comparisons with published descriptions, illustrations, and available type or reference material, supplemented by detailed morphological examinations of newly collected specimens.

## Materials and methods

### Sample collection, specimen examination, and isolation

Submerged decaying wood were collected from Dulongjiang River in Yunnan Province, China, in 2023. Decaying wood samples with a diameter of 1–2 cm and a length of approximately 20 cm were placed in plastic ziplock bags and transported to the laboratory, and incubated in plastic boxes lined with moistened tissue paper at room temperature for one week before examination (Shen et al. 2023). Macro-morphological characteristics were observed using an Optec SZ 760 stereomicroscope (Chongqing Optec Instrument Co., Ltd., Chongqing, China). Temporary microscope slides were prepared and observed under a Nikon ECLIPSE Ni-U compound microscope (Nikon Corporation, Tokyo, Japan) for detailed micro-morphological characterization and photomicrography. Colony morphology on natural substrates was photographed using a Nikon SMZ1000 stereo zoom microscope (Nikon Corporation, Tokyo, Japan).

Single-spore isolations were performed on potato dextrose agar (PDA). Conidia from target colonies were transferred onto PDA plates using a sterile toothpick. The plates were incubated at room temperature for approximately 12 hours to allow germ tube formation. Germinating conidia were then transferred to fresh PDA and incubated at room temperature. Additional germinating conidia, together with a small amount of surrounding medium, were mounted on temporary slides and photographed to document their germination characteristics.

The specimens (dry wood with fungal material) were deposited in the herbarium of Cryptogams, Kunming Institute of Botany, Chinese Academy of Sciences (HKAS), Kunming, China. Living cultures were deposited in the Kunming Institute of Botany Culture Collection Center, Kunming, China (KUNCC) and China General Microbiological Culture Collection Center (CGMCC), Beijing, China. The Fungal Names (FN) number for the newly described species was registered through the Fungal Names Repository (https://nmdc.cn/fungalnames/). The data for new taxa are lodged with the Greater Mekong Subregion microfungi database (Chaiwan et al. 2021).

### DNA extraction, PCR amplification, and sequencing

A sterile scalpel was used to scrape fresh mycelia from pure cultures growing on PDA, which were then transferred into 1.5 mL centrifuge tube (Shen et al. 2023). Genomic DNA was extracted using the Trelief™ Plant Genomic DNA Kit (TSP101-50) following the manufacturer’s instructions (Beijing Tsingke Biological Engineering Technology and Services Co., Ltd, Beijing, P.R. China). Five gene regions, LSU, ITS, *rpb*2, SSU and *tef*1-α were amplified using ITS5/ITS4 (White et al. 1990), LR0R/LR5 (Vilgalys and Hester 1990), fRPB2-5F/fRPB2-7cR, NS1/NS4 (White et al. 1990) and 983F/2218R (Liu et al. 1999), and the primer sequences are available in the WASABI database hosted on the AFTOL website (aftol.org). PCR reactions were performed in a 25 µL mixture containing 12.5 µL of 2 × GS Taq PCR MasterMix, 1 µL of each primer (forward and reverse), 9.5 µL of double-distilled water, and 1 µL of template DNA extract (Luo et al. 2018). Amplification conditions for LSU, ITS, *rpb*2, SSU and *tef*1-α were followed Shen et al. (2025). PCR products were verified on 1% agarose gels stained with ethidium bromide. Purification and sequencing were outsourced to Tsingke Biological Engineering Technology and Services Co., Yunnan, China.

### Sequence alignment and dataset preparation

Sequences were assembled using BioEdit. BLASTn searches were conducted to retrieve closely related sequences from GenBank (http://www.ncbi.nlm.nih.gov), as well as from relevant publications (Liu et al. 2025; Shen et al. 2025). Consensus and reference sequences were aligned using MAFFT v.7.0 (Katoh et al. 2019). Poorly aligned regions were trimmed with trimAl v1.2 using default parameters (Capella-Gutierrez et al. 2009). The aligned datasets for the five loci (LSU, ITS, *rpb*2, SSU and *tef*1-α) were concatenated in SequenceMatrix v.1.7.8 (Vaidya et al. 2011). Alignment formats were converted to PHYLIP and NEXUS using AliView (Larsson 2014).

### Phylogenetic analysis

Phylogenetic analyses were conducted using maximum likelihood (ML) and Bayesian tree building criteria. Maximum likelihood (ML) analysis was carried out using RAxML-HPC2 on XSEDE (8.2.12) (Stamatakis 2006; Stamatakis et al. 2008) on the CIPRES Science Gateway website (Miller et al. 2010). Maximum likelihood bootstrap support was calculated from 1000 bootstrap replicates. Bayesian analyses were carried out using MrBayes v 3.2. (Ronquist et al. 2012). The evolution model was estimated using MrModeltest 2.3, and the GTR+I+G model was the best-fit model for all gene regions. Posterior Probabilities (PP) were performed by Markov Chain Monte Carlo sampling (MCMC) in MrBayes v.3.1.2. Six simultaneous Markov chains were run for 50 million generations, and trees were sampled every 5,000 generations. The first 25% of sampled trees were discarded as burn-in, and the remaining trees were used to calculate the majority-rule consensus tree. Phylogenetic trees were visualized with FigTree v.1.4.0 and graphically edited in Adobe Illustrator (Adobe Systems Inc., USA). Newly generated sequences were deposited in GenBank (Suppl. material 1).

## Results

### Phylogenetic analysis

**Figure 1.**
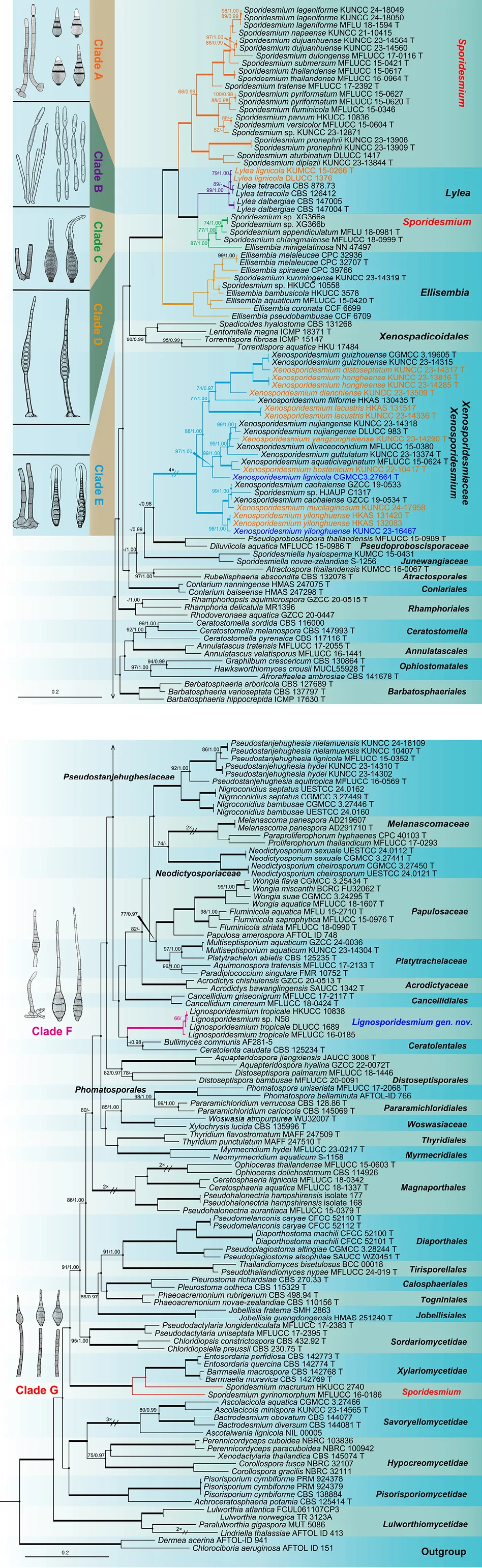
Phylogram generated from maximum likelihood analysis based on combined LSU, ITS, *rpb*2, *tef*1-α, and SSU sequences dataset representing *Sordariomycetes*. 197 strains are included in the combined analyses which comprise 4,244 characters including gaps (860 characters for LSU, 532 characters for ITS, 974 characters for SSU, 874 characters for *rpb*2, and 1,004 characters for *tef*1-α). *Chlorociboria aeruginosa* (AFTOL-ID 151) and *Dermea acerina* (AFTOL-ID 941) were selected as the outgroup taxa. Phylogenetic trees generated from maximum likelihood and Bayesian inference analyses were similar in overall topologies. Likelihood of the final tree is evaluated and optimized under GAMMA. The best RAxML tree with a final likelihood value of –125613.051159 is presented. The matrix contained 2935 distinct alignment patterns, with 32.07% undetermined characters or gaps. Estimated base frequencies were as follows: A = 0.242946, C = 0.259371, G = 0.279369, T = 0.218314; substitution rates AC = 1.223069, AG = 2.916762, AT = 1.297659, CG = 1.174808, CT = 6.365041, GT = 1.000000, α = 0.366237, Tree-Length: 19.688158. Bayesian analyses generated 8,800 trees (average standard deviation of split frequencies: 0.009914) from which 6,600 were sampled after 25% of the trees were discarded as burn-in. The alignment contained a total of 2,935 unique site patterns. Bootstrap support values with a ML greater than 65%, and Bayesian posterior probabilities (PP) greater than 0.97 are given above the nodes as MLBS/BYPP. Thickened branches represent nodes with maximum likelihood bootstrap support (MLBS) of 100% and Bayesian posterior probability (BYPP) values of 1.00. A hyphen (–) indicates values lower than 65% MLBS or 0.97 PP. Ex-type cultures are indicated by “T” following the strain numbers, newly generated sequences are shown in bold blue, and new combinations are highlighted in orange.

Maximum likelihood (ML) phylogenetic analysis was performed using a concatenated dataset comprising LSU, ITS, SSU, *rpb*2, and *tef*1-α sequences (4,244 characters, including gaps) from 195 ingroup strains, primarily representing members of the *Sordariomycetes*. The resulting phylogeny resolved *Sporidesmiumsensu lato* into seven well-supported clades (Fig. 1). Clade A, situated at the apex of the phylogenetic tree, is strongly supported and comprises 14 species along with an unnamed strain, *Sporidesmium* sp. (KUNCC 23-12871). Clade B is closely allied to Clade C and includes three species of *Lylea*, *L. dalbergiae* (CBS 147004 and CBS 147005), *L. lignicola* (KUMCC 15-0266 and DLUCC 1376) and *L. tetracoila* (CBS 878.73). Clade C comprises *Sporidesmium appendiculatum*, *S. chiangmaiense*, *Ellisembia minigelatinosa* (NN 47497), and two additional strains *Sporidesmium* sp. (XG366a and XG366b), forming a distinct, well-supported lineage sister to clade B. Clade D contains seven species previously treated as *Ellisembiasensu stricto* by Delgado et al. (2024), and it forms a strongly supported sister clade to Clades A, B and C with 100% ML and 1.00 PP support. Clade E comprises 16 species corresponding to *Xenosporidesmium (Xenosporidesmiaceae)* as circumscribed by Liu et al. (2025). Clade F consists of four strains of *Lignosporidesmium tropicale* (DLUCC 1689, HKUCC 10838, MFLUCC 16-0185 and N58), which cluster with *Bullimyces communis* (AF281-5) and *Ceratolenta caudata* (CBS 125234) of *Ceratolentales*, although this relationship lacks robust support. Clade G includes two genetically divergent species, *Sporidesmium macrurum* (HKUCC 2740) and *S. gyrinomorphum* (KUNCC 23-13816), which form a lineage nested within *Xylariomycetidae*.

### Taxonomy

#### Lignosporidesmium

Taxon classificationFungiDistoseptisporalesXenosporidesmiaceae

H.W. Shen & Z.L. Luo gen. nov.

39635431-E05B-5624-815C-5E34DA586993

Fungal Names: FN 574178

##### Chinese name.

木葚孢属 (Pinyin: mù shèn bāo shǔ).

##### Etymology.

From *ligno*- (Latin *lignum*, “wood”), referring to its wood-inhabiting habit, and *Sporidesmium*, a related hyphomycetous genus. The name indicates a sporidesmium-like fungus occurring on woody substrates.

##### Type species.

*Lignosporidesmium tropicale* (M.B. Ellis) H.W. Shen & Z.L. Luo, comb. nov.

##### Description.

***Saprobic*** on decaying wood substrates in freshwater and terrestrial habitats. ***Sexual morph***: Undetermined. ***Asexual morph***: Hyphomycetous. ***Colonies*** on superficial substratum, effuse, hairy, solitary or clustered, brown to black. ***Mycelium*** partly immersed and partly superficial, composed of septate, branched, pale to brown, smooth-walled hyphae. ***Conidiophores*** macronematous, mononematous, singly or in fascicles, unbranched, cylindrical, straight or slightly flexuous, septate, pale brown to dark brown, smooth and thick-walled. ***Conidiogenous cells*** monoblastic, terminal, integrated, percurrently proliferating, cylindrical, brown, smooth and thick-walled. ***Conidia*** acrogenous, solitary, pyriform, obclavate, rostrate, straight or slightly curved, euseptate, pale brown to dark brown, tapering to the apex, truncate at the base, thick-walled and verrucose, without sheath.

##### Notes.

Phylogenetic analyses recovered four strains of *Lignosporidesmium tropicale* (DLUCC 1689, HKUCC 10838, MFLUCC 16-0185 and N58) forming a sister clade with *Bullimyces communis* (AF281-5) and *Ceratolenta caudata* (CBS 125234) of *Ceratolentales*, although this relationship is not supported by stable statistical values. Morphologically, *Lignosporidesmium tropicale* differs from members of clades B, C, and D by possessing euseptate conidia, whereas taxa in these three clades are characterized by distoseptate conidia (Su et al. 2016; Yang et al. 2018; Luo et al. 2019; Bao et al. 2021; Crous et al. 2021b; Delgado et al. 2025; Liu et al. 2025; Zhang et al. 2025b). Furthermore, *Lignosporidesmium* is readily distinguished from species of clade A by its non-proliferating conidiogenous cells and pale brown to brown, obclavate, rostrate conidia; in contrast, species of clade A typically exhibit continuously proliferating conidiogenous cells and mature conidia that are brown to black and pyriform or lageniform (Su et al. 2016; Zhang et al. 2025a, 2025b). Phylogenetically, clades A, B, and C are placed in *Sporidesmiaceae*, clade D is accommodated in *Xenosporidesmiaceae*, whereas *Lignosporidesmium* represents an isolated lineage that cannot be assigned to any currently recognized family (Fig. 1). Therefore, based on a combination of distinctive morphological characters and phylogenetic evidence, we propose that the new genus *Lignosporidesmium* should accommodate *L. tropicale*, which forms a single, independent evolutionary lineage. The genus is herein placed in *Diaporthomycetidae* genera *incertae sedis*.

#### Lignosporidesmium tropicale

Taxon classificationFungiDistoseptisporalesXenosporidesmiaceae

(M.B. Ellis) H.W. Shen & Z.L. Luo comb. nov.

D0EEEE07-91F9-5154-9927-6ADCA7E64CD9

Fungal Names: FN 574179

≡ Sporidesmium tropicale M.B. Ellis, Mycological Papers 70: 58 (1958).

##### Chinese name.

热带木葚孢 (Pinyin: rè dài mù shèn bāo).

##### Holotype.

Sierra Leone, Njala (Kori), on branches of *Psidii guajavae*, 26 July 1949, F. C. Deighton, I.M.I. 37498 (Ellis 1985).

#### Xenosporidesmium lignicola

Taxon classificationFungiDistoseptisporalesXenosporidesmiaceae

Y. Wang, H.W. Shen & Z.L. Luo sp. nov.

6D98D494-4262-5168-99D6-B532FED6E713

Fungal Names: FN 574180

Fig. 2

##### Chinese name.

木生异葚孢 (Pinyin: mù shēng yì shèn bāo).

**Figure 2.**
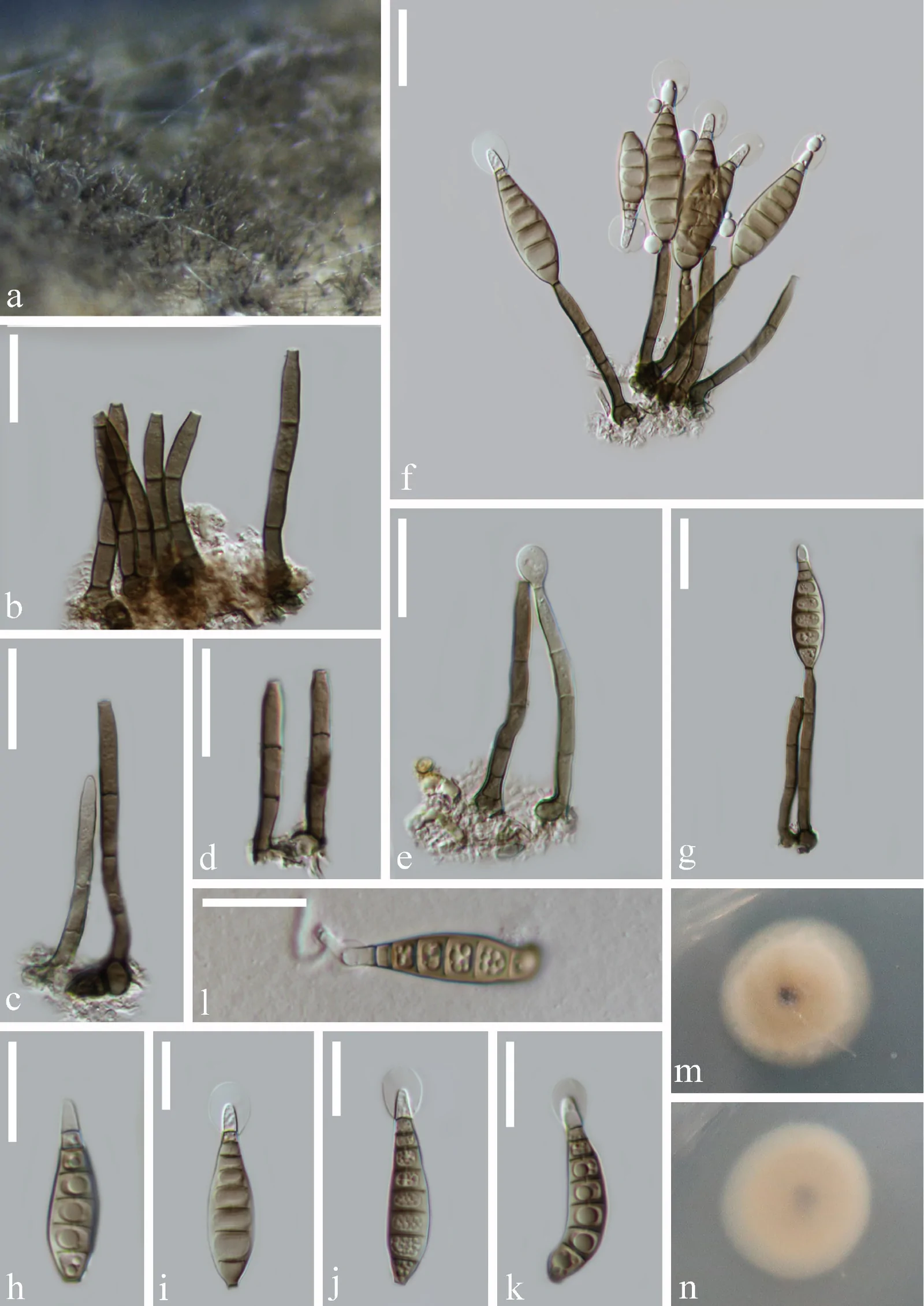
*Xenosporidesmium lignicola* (HKAS 135961, holotype). **a**. Colonies on natural substrate; **b–d**. Conidiophores; **e**. Conidiophores and developing conidium; **f, g**. Conidiophores and conidia; **h–k**. Conidia; **l**. Germinating conidium. Culture on PDA from (**m**) front and (**n**) reverse. Scale bars: 20 μm (**b–g**); 15 μm (**h–l**).

##### Etymology.

“*lignicola*” refers to the species inhabiting woody substrates.

##### Holotype.

HKAS 135961.

##### Description.

***Saprobic*** on submerged decaying wood in freshwater habitat. ***Sexual morph***: Undetermined. ***Asexual morph***: Hyphomycetous. ***Colonies*** on superficial substratum, effuse, dark olivaceous, hairy. ***Mycelium*** mostly superficial, partly immersed, composed of branched, septate, hyaline to pale brown, smooth-walled hyphae. ***Conidiophores*** (26.5–)34–49(–60) × 3.2–4.5 μm (*x̄* = 41 × 3.9 μm, n = 40) mononematous, macronematous, erect, solitary or in small groups, cylindrical, straight or flexuous, brown to olivaceous brown, 2–4-septate, mostly 3-septate, truncate at the apex and tapering towards the rounded apex, slightly swollen at the base, smooth-walled. ***Conidiogenous cells*** 12.5–16.7(–19.7) × 3.5–4.5 μm (*x̄* = 14.7 × 3.9 μm, n = 40), monoblastic, integrated, terminal, cylindrical, narrowing at the tip, brown to olivaceous brown, smooth-walled. ***Conidia*** (27–)32–40(–43) × 8–10(–12) μm (*x̄* = 36 × 9.3 μm, n = 40) acrogenous, solitary, broadly fusiform to obclavate, pale brown to olivaceous brown, hyaline at apex, straight or slightly curved, 6–8-distoseptate, rounded at the apex, truncate at the base, guttulate, smooth-walled, apical 1(–2) cells surrounded by a thin, subglobose mucilaginous sheath, 7–11 μm diam.

##### Culture characteristics.

Conidia germinating on PDA within 12 h producing germ tubes from the ends of conidium. Colonies growing on PDA for one month at room temperature in the dark. In surface view, colonies are pale yellowish-brown to beige, with a darker brown central zone, in reverse view, pale yellowish-brown to beige.

##### Material examined.

China, Yunnan Province, Dulongjiang River, 28.033444°N, 98.317358°E (elevation 1702 m), on submerged decaying wood, 1 May 2023, Ying Wang, S4446 (HKAS 135961, holotype), ex-type culture, CGMCC 3.27664 = KUNCC 23-15808.

##### Notes.

In multigene phylogenetic analyses, *Xenosporidesmium lignicola* formed a well-supported clade together with *X. aquaticivaginatum* (MFLUCC 15-0624), *X. bostenicum* (KUNCC 22-10417), *X. guttulatum* (KUNCC 23-13374), *X. nujiangense* (DLUCC 983; KUNCC 23-14318), *X. olivaceoconidium* (MFLUCC 15-0380), and *X. yangzonghaiense* (KUNCC 23-14290), but was resolved as a genetically distinct lineage (Fig. 1). Morphologically, *X. lignicola* conforms to the diagnostic characters of *Xenosporidesmium* in possessing macronematous, mononematous, cylindrical, septate conidiophores; holoblastic, monoblastic, integrated, terminal, determinate, cylindrical, smooth conidiogenous cells, occasionally proliferating percurrently; acrogenous, obclavate, pale brown to olivaceous, straight or curved, distoseptate conidia that are paler toward the apex and bear an apical appendage or a mucilaginous sheath. Nevertheless, *X. lignicola* can be distinguished from its phylogenetically closely related species by a combination of morphological characteristics, as summarized in Table 1.

**Table 1.** Currently accepted species of *Xenosporidesmium* and comparison of their main morphological characteristics.

**Taxa**	**Conidiophore**	**Conidiogenous cells**	**Conidia**	**Sheath**
*X. aquaticivaginatum*	60–125 × 4–6 µm, 1–3-septate	Occasionally proliferating	49.5–80.5 × 10.5–14 µm, obclavate, 6–10-distoseptate	Extended apical sheath (reaching mid-length)
*X. bostenicum*	22–43 × 4–7 µm, 2–3-septate	10–12 × 4–5 µm	43–106 × 8–11 µm, elongated obclavate, rostrate, 7–18-distoseptate, occasionally proliferating	Extended apical sheath (reaching mid-length)
*X. caohaiense*	25.5–50 × 3.5–6 µm, 1–3-septate	9–14.5 × 2.5–5.5 µm, occasionally proliferating	35–51 × 7.5–11 µm, obclavate, 8–13-distoseptate	Apical sheath
*X. distoseptatum*	49–61 × 3.5–5 µm, 2–3-septate	19–24 × 4–5 µm	57–85 × 9–12 µm, obclavate, rostrate, 11–16-distoseptate	Extended apical sheath (reaching mid-length, 4–8 cells)
*X. dianchiense*	47–60 × 3.7–4.8 µm, 2–3-septate	17–21 × 3.5–5 µm	46–55 × 9–11 µm, obpyriform, obclavate, rostrate, 7–9-distoseptate	Apical sheath (1–2 cells)
*X. filiforme*	22–54 × 3.5–4.9 µm, 1–3-septate	12–16 × 3.6–4.9 µm	53–100 × 5.8–9.5 µm, obclavate, mostly 10-distoseptate	Absent
*X. guizhouense*	23–59 × 2–4.6 µm, 1–5-septate	13–25 × 3.1–4 µm	46–86 × 7–11.4 µm, obclavate, obspathulate, rostrate, 9–16-distoseptate	Apical sheath
*X. guttulatum*	26–55 × 3.4–5 µm, 1–3-septate	12–20 × 3–4.8 µm	44–84 × 8.7–12 µm, obclavate, 6–14-distoseptate	Extended apical sheath (reaching mid-length)
*X. hongheense*	33–46 × 4–5 µm, 1–3-septate	17–20 × 3.5–5 µm	52–65 × 7.5–9 µm, oblong-obclavate, rostrate, 9–10-distoseptate, occasionally proliferating	Apical sheath (2–3 cells)
*X. lacustris*	40–55 × 4–5.5 µm, 1–3-septate	17–22 × 3.5–4.5 µm	49–59 × 10–13 µm, obpyriform, obclavate, rostrate, 9–11-distoseptate	Apical sheath (3–5 cells)
*X. lignicola*	34–49 × 3.2–4.5 µm, 2–4-septate	12.5–16.7 × 3.5–4.5 µm	32–40 × 8–10 µm, broadly fusiform to obclavate, 6–8-distoseptate	Apical sheath (1–2 cells)
*X. mucilaginosum*	33–72 × 3–6 µm, 2–3-septate	8–16 × 4–5 µm, occasionally proliferating	41–68 × 9–15 µm, obclavate, rostrate, 9–11-distoseptate	Extended apical sheath (reaching mid-length)
*X. nujiangense*	31–51 × 4–5.5 µm, 1–4-septate	11.5–16.5 × 4–5 µm	54–69 × 10–12.5 µm, obclavate, 10–14-distoseptate	Apical sheath
*X. olivaceoconidium*	25–55 × 3–4 µm, 1–3-septate	11–15 × 3–5 µm	25–50 × 6–10 µm, obclavate, 9–16-distoseptate	Apical sheath
*X. yangzonghaiense*	42–62 × 3–5 µm, 2–5-septate	14–18 × 3.4–5 µm	53–67 × 9–11 µm, obclavate, rostrate, 10–12-distoseptate	Extended apical sheath (5–6 cells)
*X. yilonghuense*	27–39 × 3–5 µm, 1–4-septate	11–13 × 3–4.5 µm	41–51 × 8–9 µm, obclavate, rostrate, 7–9-distoseptate	Apical sheath (1–3 cells)

#### Xenosporidesmium yilonghuense

Taxon classificationFungiDistoseptisporalesXenosporidesmiaceae

(H.W. Shen & Z.L. Luo) H.W. Shen & Z.L. Luo comb. nov.

A0FAB662-64D2-5C02-8E1C-3001380F8AB5

Fungal Names: FN 74189

Fig. 3

≡ Sporidesmium yilonghuense H.W. Shen & Z.L. Luo, Fungal Diversity 134: 853 (2025).

##### Chinese name.

异龙湖异葚孢 (Pinyin: yì lóng hú yì shèn bāo).

**Figure 3.**
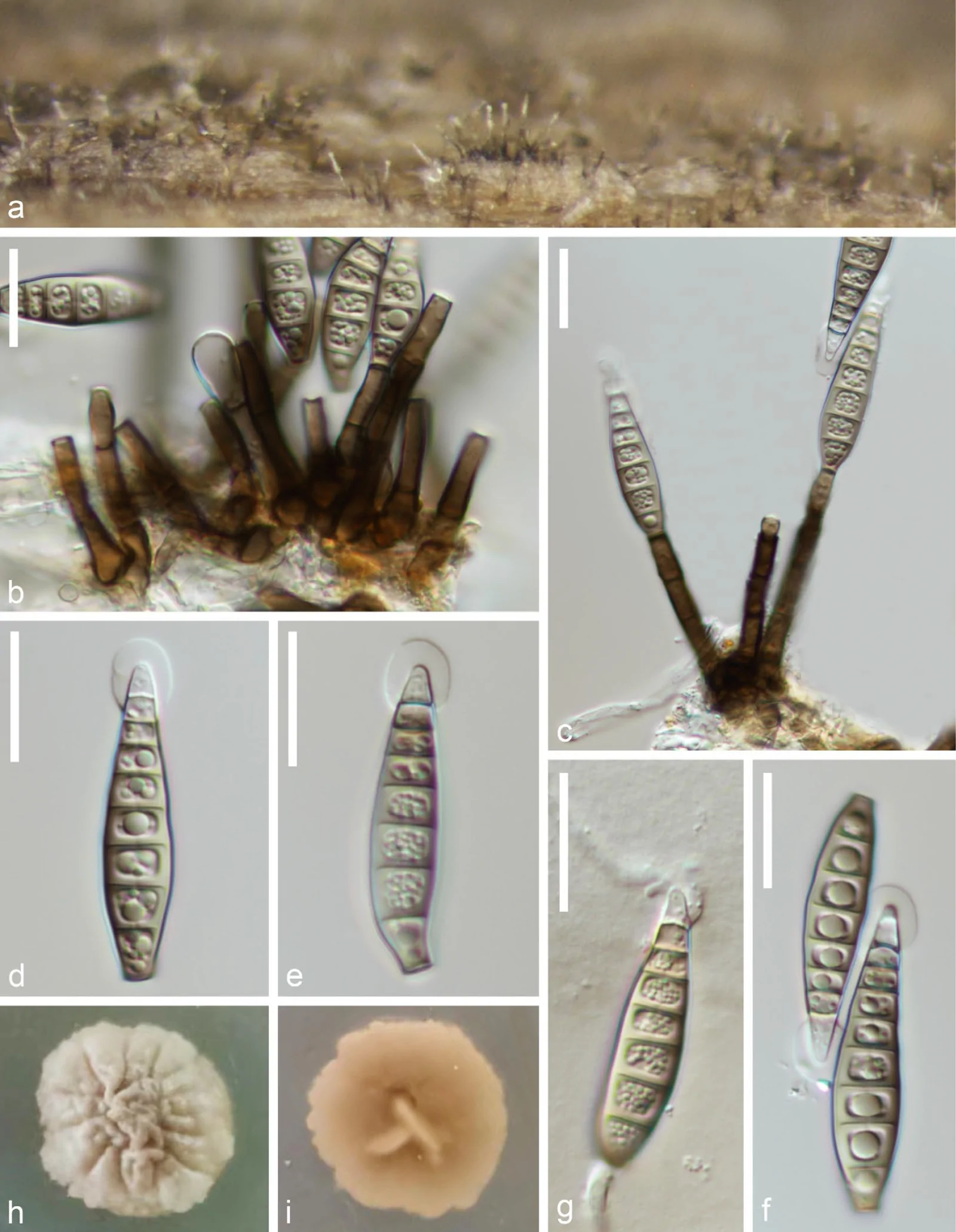
*Xenosporidesmium yilonghuense* (HKAS 135964). **a**. Colonies on natural substrate; **b, c**. Conidiophores, conidiogenous cells and conidia; **d–f**. Conidia; **g**. Germinating conidium. Culture on PDA from (**h**) front and (**i**) reverse. Scale bars: 15 μm (**b–g**).

##### Description.

***Saprobic*** on submerged decaying wood in freshwater habitats. ***Sexual morph***: Undetermined. ***Asexual morph***: Hyphomycetous. ***Colonies*** on superficial substratum, effuse, dark brown, hairy. ***Mycelium*** mostly superficial, partly immersed, composed of branched, septate, hyaline to pale brown, smooth-walled hyphae. ***Conidiophores*** (16–)21–40(–54) × 3.5–5 μm (*x̄* = 31 × 4 μm, n = 40), macronematous, mononematous, solitary or fascicular, cylindrical, straight to slightly flexuous, brown to dark brown, 2–4-septate, slightly swollen at the base, truncate at the apex, smooth-walled, sometimes percurrently proliferating. ***Conidiogenous cells*** 9–14 × 3–4.7 μm (*x̄* = 11 × 3.8 μm, n = 30), holoblastic, monoblastic, terminal, integrated or percurrently proliferating, cylindrical, slightly narrowing at the tip, brown, smooth-walled. ***Conidia*** (28–)33–40(–43) × 7.5–9 μm (*x̄* = 36.5 × 8.4 μm, n = 30), acrogenous, solitary, obclavate, pale brown to brown, hyaline at apex, straight or slightly curved, 7–9‐distoseptate, truncate at the base, rounded at the apex, guttulate, smooth‐walled, apical 1–2 cells surrounded by a mucilaginous sheath.

##### Culture characteristics.

Conidia germinated on PDA within 24 h. Germ tubes produced from the basal cell of conidia. Colonies growing on PDA for one month at room temperature in the dark. Circular, wrinkled, white to pale yellow from above, yellow brown and paler towards the margin from below.

##### Material examined.

China, Yunnan Province, Dulongjiang River, 28.033444°N, 98.317358°E (elevation 1702 m), on submerged decaying wood, 1 May 2023, Ying Wang, S5266 (HKAS 135964), living culture, KUNCC 23-16467.

##### Notes.

Phylogenetic analyses showed that the newly collected strain, KUNCC 23-16467, clustered stably with previously reported strains of *X. yilonghuense* (HKAS 131420 and HKAS 132083) collected from freshwater lakes (Fig. 1). *Xenosporidesmium yilonghuense* has been reported from unknown submerged decaying wood in Dujuanhu Lake and Yilonghu Lake, Yunnan Province, China (Shen et al. 2025). In the present study, strain, KUNCC 23-16467, was re-collected from a lotic freshwater habitat. Based on detailed morphological comparisons and multi-locus phylogenetic analyses, this strain was identified as *X. yilonghuense*. Morphologically, the new collection is characterized by macronematous, mononematous, cylindrical conidiophores that are 2–4-septate; conidiogenous cells are holoblastic, monoblastic, terminal, and integrated; and conidia are acrogenous, solitary, obclavate, and distoseptate, with the apical 1–2 cells surrounded by a mucilaginous sheath. These characters are consistent with the original description of *X. yilonghuense* (Shen et al. 2025). Molecular sequence comparisons revealed nucleotide differences of 13 bp (ITS), 9 bp (LSU), and 2 bp (*tef*1-α) between strain KUNCC 23-16467 and the type material HKAS 131420. Therefore, based on morphological congruence and phylogenetic placement, the newly collected specimen is confirmed as *X. yilonghuense*. This new record extends the known distribution of *X. yilonghuense* and provides additional morphological and molecular data supporting species delimitation within *Xenosporidesmium*.

### Other new combinations

Based on phylogenetic analyses and morphological characteristics, the following new combinations are proposed for recently published species:

#### Lylea lignicola

Taxon classificationFungiDistoseptisporalesXenosporidesmiaceae

(Z.L. Luo, K.D. Hyde & H.Y. Su) H.W. Shen & Z.L. Luo comb. nov.

27874830-9159-5FD6-A3A6-D70EE26B3E1E

Fungal Names: FN 74181

≡ Sporidesmium lignicola Z.L. Luo, K.D. Hyde & H.Y. Su, Fungal Diversity 99: 507 (2019).

##### Chinese name.

木生莱尔孢 (Pinyin: mù shēng lái ěr bāo).

#### Xenosporidesmium bostenicum

Taxon classificationFungiDistoseptisporalesXenosporidesmiaceae

(R.J. Xu, Q. Zhao & K.D. Hyde) H.W. Shen & Z.L. Luo comb. nov.

0DC41F1D-4455-5AD2-A355-523A3CD91444

Fungal Names: FN 74182

≡ Sporidesmium bostenicum R.J. Xu, Q. Zhao & K.D. Hyde, Fungal Diversity 133: 175 (2025).

##### Chinese name.

博斯腾异葚孢 (Pinyin: bó sī téng yì shèn bāo).

#### Xenosporidesmium dianchiense

Taxon classificationFungiDistoseptisporalesXenosporidesmiaceae

(H.W. Shen & Z.L. Luo) H.W. Shen & Z.L. Luo comb. nov.

6F1EDE6A-97CA-5CB8-A2AF-EBB298222B07

Fungal Names: FN 74183

≡ Sporidesmium dianchiense H.W. Shen & Z.L. Luo, Fungal Diversity 134: 841 (2025).

##### Chinese name.

滇池异葚孢 (Pinyin: diān chí yì shèn bāo).

#### Xenosporidesmium distoseptatum

Taxon classificationFungiDistoseptisporalesXenosporidesmiaceae

(H.W. Shen & Z.L. Luo) H.W. Shen & Z.L. Luo comb. nov.

5BCD8418-9B0E-57EF-8B7C-5F4E3C252C36

Fungal Names: FN 74184

≡ Sporidesmium distoseptatum H.W. Shen & Z.L. Luo, Fungal Diversity 134: 841 (2025).

##### Chinese name.

假隔异葚孢 (Pinyin: jiǎ gé yì shèn bāo).

#### Xenosporidesmium hongheense

Taxon classificationFungiDistoseptisporalesXenosporidesmiaceae

(H.W. Shen & Z.L. Luo) H.W. Shen & Z.L. Luo comb. nov.

0120E1CF-F141-50C5-958F-7E4C772394AD

Fungal Names: FN 74185

≡ Sporidesmium hongheense H.W. Shen & Z.L. Luo, Fungal Diversity 134: 845 (2025).

##### Chinese name.

红河异葚孢 (Pinyin: hóng hé yì shèn bāo).

#### Xenosporidesmium lacustris

Taxon classificationFungiDistoseptisporalesXenosporidesmiaceae

(H.W. Shen & Z.L. Luo) H.W. Shen & Z.L. Luo comb. nov.

55A1584F-3D7A-5F90-906F-59AC43716226

Fungal Names: FN 74186

≡ Sporidesmium lacustris H.W. Shen & Z.L. Luo, Fungal Diversity 134: 847 (2025).

##### Chinese name.

湖泊异葚孢 (Pinyin: hú pō yì shèn bāo).

#### Xenosporidesmium mucilaginosum

Taxon classificationFungiDistoseptisporalesXenosporidesmiaceae

(R.J. Xu, Q. Zhao & K.D. Hyde) H.W. Shen & Z.L. Luo comb. nov.

4010A8F8-5BCA-5C30-8BFB-2275052353D3

Fungal Names: FN 74187

≡ Sporidesmium mucilaginosum R.J. Xu, Q. Zhao & K.D. Hyde, Fungal Diversity 133: 177 (2025).

##### Chinese name.

粘鞘异葚孢 (Pinyin: nián qiào yì shèn bāo).

#### Xenosporidesmium yangzonghaiense

Taxon classificationFungiDistoseptisporalesXenosporidesmiaceae

(H.W. Shen & Z.L. Luo) H.W. Shen & Z.L. Luo comb. nov.

30A1A568-ED05-5E6D-931A-30F4094E0204

Fungal Names: FN 74188

≡ Sporidesmium yangzonghaiense H.W. Shen & Z.L. Luo, Fungal Diversity 134: 850 (2025).

##### Chinese name.

阳宗海异葚孢 (Pinyin: yáng zōng hǎi yì shèn bāo).

## Discussion

Phylogenetic analyses of *Sporidesmium*-related taxa based on a combined dataset of LSU, ITS, *rpb*2, SSU and *tef*1-α sequence data, resolved seven distinct lineages (Fig. 1). Clade A comprises a well-supported monophyletic group of 14 sequenced species characterized by macronematous, mononematous, unbranched, cylindrical, conidiophores; monoblastic, integrated, determinate or percurrently proliferating conidiogenous cells; and brown to dark brown, euseptate, pyriform, lageniform, obclavate, rostrate conidia with hyaline apical cells. Morphologically, members of this clade correspond closely to *Sporidesmium atrum* as described and illustrated in paper of Ellis (1985). However, because the type specimen of *S. atrum* has been lost, a specimen annotated as “*Sporidesmium fusiforme* Nees ex Esenb. — *Sporidesmium atrum* Link var. teste Link”, which was subsequently shown not to represent *S. fusiforme*, was described as *S. ehrenbergii* and designated as the lectotype of *Sporidesmium* (Ellis 1985; Su et al. 2016). *Sporidesmium ehrenbergii* is characterized by macronematous, mononematous, unbranched, cylindrical, septate conidiophores; monoblastic, integrated conidiogenous cells, sometimes with 1–2 proliferations; and reddish brown, obclavate to subfusiform, euseptate or distoseptate conidia (Ellis 1985). Taken together with the illustrations and descriptions, these diagnostic characters are clearly distinct from those observed in species of Clade A. Consequently, if *S. ehrenbergii* represents *Sporidesmiumsensu stricto*, Clade A should be regarded as a distinct taxonomic entity. Unfortunately, *S. ehrenbergii* has not been recollected and no molecular sequence data are currently available. As a result, its precise taxonomic position and its relationship to members of Clade A remain unresolved. In the absence of additional morphological or molecular evidence, no taxonomic revisions are proposed for Clade A, however we propose that Clade A should represent *Sporidesmiumsensu stricto*.

Clade B forms a well-supported monophyletic lineage comprising three species, *viz*., *Lylea dalbergiae*, *L. lignicola* (≡ *Sporidesmium lignicola*) and *L. tetracoila* (Fig. 1). Species of *Lylea* are characterized by micronematous to semi-macronematous, cylindrical, septate conidiophores; monoblastic, integrated, determinate conidiogenous cells; and acrogenous, catenate, straight to slightly curved, cylindrical, distoseptate, guttulate conidia with obtuse ends (Morgan-Jones 1975; Luo et al. 2019; Crous et al. 2021a). These characters, particularly the catenate conidia, clearly depart from the traditional morphological concept of *Sporidesmium* (Ellis 1985; Su et al. 2016). *Sporidesmium lignicola* was originally established primarily on the basis of phylogenetic evidence (Luo et al. 2019). Morphologically, it exhibits clustered conidiophores and catenate conidia, its features closely resembling those of *Lylea tetracoila*, although the latter has longer conidia (Koukol and Delgado 2021). Based on these similarities, Koukol and Delgado (2021) regarded *S. lignicola* and *L. tetracoila* as conspecific and treated them under the name *Sporidesmium tetracoilum*. Crous et al. (2021a) introduced *Lylea dalbergiae*, which conforms well to the generic concept of *Lylea*, having micronematous, solitary to aggregated, inconspicuous, subcylindrical conidiophores; monoblastic, integrated, terminal, subcylindrical conidiogenous cells; and acrogenous, catenate, subcylindrical, distoseptate, guttulate conidia with subobtuse ends. In the phylogenetic analyses, this species clusters with *L. lignicola* (DLUCC 1376 and KUMCC 15-0266) and *L. tetracoila* (CBS 878.73 and CBS 126412). Based on both phylogenetic placement and diagnostic morphological characters, we consider *Sporidesmium tetracoilum* and *Lylea dalbergiae* to be inconsistent with *Sporidesmium* and therefore support the resurrection of *Lylea tetracoila*. However, whether *Sporidesmium lignicola* and *Lylea tetracoila* represent the same species remains uncertain, as molecular data for the type specimen of *L. tetracoila* are currently unavailable. Furthermore, specimens identified as *L. tetracoila* have thus far been reported only from Europe and Canada, and all available sequence data originate from European herbarium material (Shenoy et al. 2006; Koukol and Delgado 2021). In the present study, we generated the ITS sequence for the ex-paratype of *S. lignicola* (DLUCC 1376). BLASTn searches revealed that the closest match to *S. lignicola* (DLUCC 1376) was *Lylea tetracoila* (CBS 126412) (GenBank accession MH864106; 505/513 bp identical, 98% identity, one gap). Considering both the limited molecular evidence and the apparent geographical separation, we tentatively recognize *Sporidesmium lignicola* as a species distinct from *Lylea tetracoila* and accordingly transfer it to *Lylea* as *L. lignicola*.

*Sporidesmium appendiculatum* and *S. chiangmaiense* were collected from submerged driftwood in a stream in Chiang Mai Province, Thailand (Dong et al. 2021). Morphologically, both species conform to the diagnostic features of *Sporidesmiumsensu lato* (Ellis 1985; Su et al. 2016). Phylogenetic analyses placed these two species in a well-supported clade together (Clade C) with *Ellisembia minigelatinosa* (NN 47497) and two unnamed strains of *Sporidesmium* sp. (XG366a and XG366b), forming a distinct lineage that is sister to clade B (Fig. 1). Based on the current phylogenetic framework, this lineage represents an independent evolutionary lineage distinct from *Sporidesmiumsensu stricto*. Careful examination of the original descriptions and illustrations indicates that *S. appendiculatum* and *S. chiangmaiense* share a distinctive conidial morphology, characterized by a cuneiform basal cell that is darker in color (brown to dark brown) than the remaining conidial cells, in combination with a filamentous appendage and a gelatinous, hyaline sheath (Dong et al. 2021). Detailed descriptions and illustrations of *Ellisembia minigelatinosa* (specimen NN 47497) were sought to assess whether this species exhibits comparable dark, cuneiform basal cells; however, such information was unavailable. Consequently, it remains unclear whether this distinctive basal cell morphology constitutes a synapomorphic character distinguishing this lineage from other *Sporidesmium*-related taxa lineages, and this hypothesis requires validation based on additional material. Although the phylogenetic analyses suggest that this lineage may represent a taxonomically distinct group separate from *Sporidesmiumsensu stricto*, further studies integrating additional morphological observations, molecular data, and representative collections are required to confirm its taxonomic status. Therefore, no formal taxonomic revision is proposed for this lineage in the present study.

Based on combined phylogenetic and morphological evidence, Delgado et al. (2024) circumscribed *Ellisembia* as a monophyletic lineage within *Sporidesmiaceae*, including the type species *E. coronata* (epitype: PRC 9257). This lineage is primarily characterized by distoseptate conidia produced from conidiophores exhibiting none or a few percurrent extensions, and the conidia lack appendages and a mucilaginous sheath (Delgado et al. 2024). However, aside from the presence of distoseptate conidial septation, these features do not markedly differ from those observed in *Sporidesmium ehrenbergii*, the lectotype species of *Sporidesmium* (Ellis 1985; Su et al. 2016), in which conidial septation is known to include both euseptate and distoseptate types (Ellis 1985). Current phylogenetic analyses indicate that *Sporidesmium* species possessing exclusively euseptate conidia are confined to clade A. Nevertheless, based on the morphological features of taxa within this clade, the traditional concept derived from *S. ehrenbergii* appears to correspond more closely with the present circumscription of *Ellisembia*. Although Delgado et al. (2024) provided a comprehensive treatment of extant *Ellisembia* species, including the designation of an epitype and the generation of molecular sequence data for *E. coronata*, further studies are still required to determine the precise taxonomic position of *Sporidesmium ehrenbergii* and to resolve its relationship with *Ellisembia* and *Sporidesmiumsensu stricto*.

*Xenosporidesmium*, established by Liu et al. (2025) based on morphological evidence and multigene phylogenetic analyses, forms a distinct lineage within *Diaporthomycetidae* and is separated from related taxa by substantial genetic divergence (Fig. 1). Morphologically, *Xenosporidesmium* conforms to the concept of *Sporidesmiumsensu lato* and shows close similarity to species in clade C. However, members of *Xenosporidesmium* are distinguished by basal conidial cells that are concolorous with the remaining conidial cells (Liu et al. 2019, 2025; Shen et al. 2025; Xu et al. 2025). In addition, the genus is readily separable from other *Sporidesmium*-related taxa lineages by the presence of a distinct, hyaline, mucilaginous sheath enclosing the apical cells (Liu et al. 2019, 2025; Shen et al. 2025; Xu et al. 2025). Although no reference sequence data are currently available for *S. ehrenbergii*, species of *Xenosporidesmium* are morphologically clearly distinguishable from other *Sporidesmium*-related taxa lineages, with the exception of clade C. Therefore, based on the combined morphological distinctions and phylogenetic evidence, we follow the treatment of Liu et al. (2025) and transfer the eight species previously described in *Sporidesmium* to *Xenosporidesmium*.

*Sporidesmium tropicale* occurs widely on diverse plant substrates in tropical regions and has also been reported from Hainan, Guangdong, and Guangxi provinces of China (Ellis 1985; Wu and Zhuang 2005; Yang et al. 2018; Bao et al. 2021; Zhang et al. 2025b). Morphologically, it is characterized by macronematous, mononematous, unbranched, cylindrical conidiophores; monoblastic, terminal, integrated conidiogenous cells, occasionally proliferating percurrently; and obclavate, rostrate, euseptate, verrucose conidia lacking mucilaginous sheaths (Ellis 1985; Wu and Zhuang 2005; Yang et al. 2018; Bao et al. 2021; Zhang et al. 2025b). The type specimen (I.M.I. 37498) currently lacks usable sequence data, and the earliest available sequence from a Chinese specimen (HKUCC 10838), for which no corresponding description or illustrations are extant. Subsequently, some specimens of this species have been collected from various freshwater and terrestrial plant substrates in China and Thailand (Bao et al. 2021; Lu et al. 2025; Zhang et al. 2025b). *Sporidesmium tropicale* appears to exhibit strong ecological adaptability, as strains from different geographical regions and habitats consistently share key morphological traits, indicating a distinct lineage with high evolutionary stability (Fig. 1). Unlike other *Sporidesmium*-related lineages, its conidia possess verrucose, a long and slender apex, and euseptate septa (Bao et al. 2021; Lu et al. 2025; Zhang et al. 2025b). Phylogenetic analyses indicate that *S. tropicale* is distantly related to *Ceratolentales* and clearly separated from other *Sporidesmium*-related taxa lineages. Based on combined morphological and phylogenetic evidence, we consider *S. tropicale* to represent a lineage distinct from *Sporidesmium*. To avoid confusion with other *Sporidesmium*-related lineages (clades A, C, and D), we therefore propose a new genus, *Lignosporidesmium*, to accommodate this lineage.

*Sporidesmium gyrinomorphum* and *S. macrurum* are related with *Xylariomycetidae* (Fig. 1). *Sporidesmium gyrinomorphum* was collected from submerged decaying wood in freshwater streams in Thailand (Yang et al. 2018). Morphologically, it is characterized by macronematous, mononematous, cylindrical, septate, dark brown conidiophores, occasionally proliferating percurrently from the apex; monoblastic, integrated, terminal, cylindrical conidiogenous cells; and acrogenous, solitary, obclavate to obspathulate, rostrate, euseptate, guttulate conidia, mid- to dark brown and without sheath (Yang et al. 2018). Overall, *S. gyrinomorphum* conforms to the concept of *Sporidesmiumsensu lato* (Ellis 1985; Su et al. 2016). However, careful examination of the illustrations suggests that its conidiogenesis differs from typical *Sporidesmiumsensu lato* species. Specifically, *S. gyrinomorphum* appears to exhibit monotretic conidiogenesis, whereas *Sporidesmiumsensu lato* is generally monoblastic (Ellis 1985; Wu and Zhuang 2005; Su et al. 2016). Due to some limitations, the type specimen of this species was not examined, and its detailed morphological characteristics and precise taxonomic placement will be addressed in future studies. Similarly, no specimens or descriptions of *Sporidesmium macrurum* were available. In addition, no specimens or descriptions of *S. macrurum* (HKUCC 2740) were available in public databases. Nevertheless, phylogenetic analyses indicate that both *S. gyrinomorphum* and *S. macrurum* are clearly distinct from other *Sporidesmium*-related taxa lineages and likely represent a separate genus. As their precise positions within *Xylariomycetidae* have not been analyzed in detail, no formal taxonomic revisions are proposed in the present study.

Overall, *Sporidesmium*-related taxa encompass several groups exhibiting either pronounced or subtle morphological differences (Ellis 1985; Wu and Zhuang 2005; Shenoy et al. 2006; Su et al. 2016; Liu et al. 2025; Lu et al. 2025; Shen et al. 2025; Xu et al. 2025; Zhang et al. 2025b). While some groups have been well-classified and standardized based on phylogenetic evidence, this applies primarily to species for which molecular sequence data are available (Su et al. 2016; Delgado et al. 2024; Liu et al. 2025). Accurate classification remains extremely challenging for species described in earlier publications or lacking sequence data, particularly when specimens are poorly preserved or incomplete. In many cases, original descriptions are brief or lack detailed illustrations, further complicating attempts to place these species reliably within the current phylogenetic framework (Ellis 1985; Wu and Zhuang 2005; Su et al. 2016). In addition, for individual researchers seeking to examine type specimens, access can be a major obstacle. Type specimens are often dispersed across multiple countries, institutions, or private herbaria, and logistical, legal, or financial constraints may hinder their study. Consequently, revising the taxonomy of *Sporidesmium*-related taxa frequently requires a combination of morphological re-examination, field recollection of fresh material, and generation of new molecular data. These challenges collectively contribute to the slow progress in achieving a comprehensive and stable classification for this morphologically diverse genus and its related lineages.

## Supplementary Material

XML Treatment for Lignosporidesmium

XML Treatment for Lignosporidesmium tropicale

XML Treatment for Xenosporidesmium lignicola

XML Treatment for Xenosporidesmium yilonghuense

XML Treatment for Lylea lignicola

XML Treatment for Xenosporidesmium bostenicum

XML Treatment for Xenosporidesmium dianchiense

XML Treatment for Xenosporidesmium distoseptatum

XML Treatment for Xenosporidesmium hongheense

XML Treatment for Xenosporidesmium lacustris

XML Treatment for Xenosporidesmium mucilaginosum

XML Treatment for Xenosporidesmium yangzonghaiense

## Additional information

### Artificial Intelligence (AI) use

The authors accept full responsibility for the content of the manuscript, including the disclosure of any use of AI.

No AI tools were used in the preparation of this manuscript.

### Author ORCIDs

H.-W. Shen https://orcid.org/0000-0003-2508-1970

K.D. Hyde https://orcid.org/0000-0002-2191-0762

Y. Wang https://orcid.org/0009-0004-1995-1671

L. Yang https://orcid.org/0000-0002-3173-8804

X.-J. Su https://orcid.org/0000-0002-6357-7750

Y.-X. Li https://orcid.org/0009-0007-5645-8861

Z.-L. Luo https://orcid.org/0000-0001-7307-4885

### Data availability

All of the data that support the findings of this study are available in the main text or Supplementary Information.

## Supplementary materials

Supplementary material 1 Taxa used in the phylogenetic analyses and their corresponding GenBank accession numbers Data type xlsx https://binary.pensoft.net/file/1824130This dataset is made available under the Open Database License (http://opendatacommons.org/licenses/odbl/1.0/). The Open Database License (ODbL) is a license agreement intended to allow users to freely share, modify, and use this Dataset while maintaining this same freedom for others, provided that the original source and author(s) are credited. Hong-Wei Shen, Kevin D. Hyde, Ying Wang, Lin Yang, Xi-Jun Su, Yun-Xia Li, Zong-Long Luo
